# OCT‐guided PCI in elderly patients

**DOI:** 10.1002/agm2.12381

**Published:** 2024-12-11

**Authors:** Yanwen Fang, Mengyue Yu

**Affiliations:** ^1^ Department of Cardiology, Fuwai Hospital, National Center for Cardiovascular Diseases Chinese Academy of Medical Sciences and Peking Union Medical College Beijing China

According to the latest official report, the incidence and mortality rates of cardiovascular diseases continue to rise in China.^1^ Among these, coronary heart disease (CHD) represents the most significant threat to public health and imposes substantial social burdens. Percutaneous coronary intervention (PCI) has rapidly advanced as a key treatment modality for CHD. With an aging population, the number of PCIs performed in China is projected to increase over the long term. To enhance the prognosis for patients with CHD, it is essential to improve the quality of care while ensuring a reasonable growth in the volume of procedures. In recent years, intravascular imaging‐guided PCI has emerged as a crucial approach for the precise optimization of PCI procedures in clinical practice. This imaging primarily encompasses intravascular ultrasound (IVUS) and optical coherence tomography (OCT). Since the 1990s, a wealth of clinical evidence has been published regarding the efficacy and safety of IVUS.^2^, ^3^ However, despite OCT's superior image resolution, high‐quality evidence concerning its impact on patient prognosis remains limited due to its more recent application. Results from two clinical trials comparing OCT‐guided PCI with angiography‐guided PCI were presented at the European Society of Cardiology Annual Meeting 2023 and subsequently published in the New England Journal of Medicine. The OCTOBER study demonstrated that OCT‐guided PCI enhances clinical outcomes for complex bifurcation lesions.^4^ Conversely, the ILUMIEN IV study reported negative findings. Although OCT‐guided PCI achieved a larger postoperative minimum stent area (MSA) in clinically high‐risk patients and/or high‐risk coronary lesions, there was no significant difference in the incidence of target vessel failure (TVF) at 2 years between the two groups.^5^ These contrasting results invite a deeper examination of the prognostic value of OCT‐guided PCI. Therefore, this article provides a comprehensive review of the clinical significance and application of OCT‐guided PCI in elderly patients, informed by an analysis of these two trials.

To effectively interpret randomized controlled trials (RCTs) and integrate their findings into clinical practice, adherence to the “PICOS principle,” which encompasses Population, Intervention, Comparison, Outcome, and Study design, is crucial. An analysis of the two clinical trials presented in Table 1 highlights that both studies are direct RCTs assessing the effectiveness of OCT compared to angiography‐guided PCI and share similar definitions for their primary end points. However, the inclusion criteria exhibit notable differences. The OCTOBER study is dedicated to validating the use of OCT specifically in true bifurcation lesions with clear indications for PCI. In contrast, the ILUMIEN IV study considers a more diverse population with a wider range of lesions. Understanding that patients with high‐risk profiles and complex lesions might derive greater benefits from OCT‐guided PCI, the ILUMIEN IV study incorporates individuals exhibiting high clinical and/or coronary artery lesion risks. High clinical risk is categorized by diabetic patients undergoing drug treatment, while high coronary artery lesion risk includes factors such as recent myocardial infarction, anticipated stent lengths exceeding 28 mm, bifurcation lesions necessitating two stents, severe calcification, chronic total occlusion, or diffuse and multifocal in‐stent restenosis. Unlike the OCTOBER study, which examined all bifurcated lesions, nearly 70% of the coronary lesions in the ILUMIEN IV study were either long or multiple lesions, with bifurcated lesions representing only about 3%. This disparity in lesion characteristics indicates that the negative outcomes of the ILUMIEN IV study do not contradict the positive results observed in the OCTOBER study, thus providing robust evidence for the clinical adoption of OCT‐guided PCI in the treatment of bifurcated lesions.

**TABLE 1:** Comparison of two trials.

	The OCTOBER study	The ILUMIEN IV study
Population	Patients with bifurcation lesions	Patients with high clinical and/or high coronary artery lesion risk
Average age	65.6 years	66.3 years
Diabetes	16.7%	42.0%
Bifurcation lesions	100%	3.3%
Long or multiple lesions*	Unknown	67.4%
Intervention	OCT‐guided PCI	OCT‐guided PCI
Comparison	Angiography‐guided PCI	Angiography‐guided PCI
Primary end point	MACE**	MSA and TVF***
Median follow‐up	2 years	2 years
Study design	Multicenter RCT	Multicenter RCT

Abbreviations: MACE, major adverse cardiac events; MSA, minimum stent area; OCT, optical coherence tomography; PCI, percutaneous coronary intervention; RCT, randomized controlled trials; TVF, target vessel failure.

* Expected stent length longer than 28 mm.

** A composite of cardiac cause, target‐lesion myocardial infarction, or ischemia‐driven target‐lesion revascularization.

*** A composite of death from cardiac causes, target‐vessel myocardial infarction, or ischemia‐driven target‐vessel revascularization.

The ILUMIEN IV study was developed following the positive outcomes of the ILUMIEN III study, which conducted a randomized comparison of coronary angiography, IVUS, and OCT‐guided PCI. This prior investigation demonstrated that the strategy of stent optimization using OCT was safe, with the postoperative MSA achieved through OCT being comparable to that obtained with IVUS, particularly concerning primary efficacy end points.^6^ Moreover, findings from an earlier study indicated that a larger postoperative MSA resulting from IVUS‐guided PCI is the most significant predictor of preventing TVF within a 2‐year period.^7^ Consequently, the co‐primary end points for the ILUMIEN IV study were established to evaluate both the primary imaging end point, MSA, and the key clinical end point, TVF, to determine whether OCT‐guided PCI could yield a greater postoperative MSA and potentially enhance clinical outcomes. However, the results revealed that, although the OCT group attained a larger postoperative MSA, this did not lead to a statistically significant reduction in the occurrence of clinical end point events. This observation challenges earlier findings that associated MSA with improved clinical outcomes. The researchers posited that the inability to meet the clinical end point in the ILUMIEN IV study might be influenced by the COVID‐19 pandemic. Despite the inclusion of patients with diabetes or high‐risk coronary artery lesions, the rates of ischemia‐driven target vessel revascularization in both the OCT and angiography groups were relatively low (5.6%) when compared to other clinical trials. This discrepancy may be explained by the constraints on medical resources during the pandemic and the difficulties in ensuring timely revascularization as symptoms arose.

Coronary artery lesions in elderly patients are generally more intricate compared to those observed in younger individuals. Such complexity is often a result of cumulative exposure to various risk factors over time, coupled with the physiological changes associated with aging.^8^ In the OCTOBER study, the mean age of participants was recorded at 65.6 years, while the ILUMIEN IV study reported a mean age of 66.3 years, indirectly highlighting this trend of increasing complexity in lesions among older populations. The findings from the OCTOBER study advocate for the application of OCT in guiding PCI specifically for bifurcated lesions, deeming it a suitable strategy. Although the ILUMIEN IV study did not meet its primary clinical end point, the advantages related to achieving a larger MSA remain significant and worthy of attention. Considering the rapid aging population in China, employing OCT to optimize PCI for complex and high‐risk lesions is expected to yield considerable benefits. Future investigations should prioritize the identification of patient demographics most likely to benefit from this approach, particularly focusing on the characteristics of their coronary artery lesions.

It is important to highlight that the prevalence of left main coronary artery (LMCA) lesions is notably high among elderly patients. IVUS is a well‐established imaging modality with substantial evidence supporting its use in guiding PCI for LMCA lesions,^9^, ^10^, ^11^ and it is endorsed by various domestic and international guidelines as well as expert consensus.^12^, ^13^ Conversely, the evidence regarding the application of OCT in LMCA lesions remains limited, and concerns regarding its use in this context persist. Firstly, OCT imaging requires the flushing of a contrast agent through the guide catheter to displace blood. The large diameter of the LMCA necessitates that operators possess advanced flushing techniques to obtain clear images without inflicting significant damage to the coronary artery or inducing cardiac ischemia. Secondly, accurately positioning the guiding catheter can pose challenges when the lesion is located at the opening of the LMCA, complicating the completion of the OCT imaging process. Nevertheless, a prospective multicenter study published in 2021 demonstrated the safety and efficacy of OCT in optimizing PCI strategies for LMCA lesions.^14^ The high resolution of OCT offers distinct advantages in assessing plaque composition and stent expansion, suggesting that LMCA lesions should not be deemed off‐limits for OCT. However, meticulous attention must be given to the selection and coaxial alignment of the guide catheter, and the procedure should be conducted by experienced interventional cardiologists. It is noteworthy that the proportion of LMCA bifurcation lesions in the OCTOBER study was only 18.9%, which is lower than the initially planned percentage. Consequently, additional clinical evidence is required to establish the safety and effectiveness of OCT in guiding interventions for left main bifurcation lesions.

In addition to the concerns regarding LMCA lesions, it is imperative to address the risk of kidney injury associated with the use of contrast agents. OCT‐guided PCI typically necessitates a higher dosage of contrast agent. In the ILUMIEN IV study, the average dosage of contrast agent in the OCT group was 231.9 ± 88.2 mL, in contrast to 198.3 ± 81.7 mL in the control group. In elderly patients, both the volume of the kidneys and renal blood flow gradually decrease, resulting in a decline in glomerular filtration rate. Consequently, the incidence of contrast‐induced acute kidney injury (CI‐AKI) is significantly elevated in elderly individuals compared to younger patients. Furthermore, impaired renal function, diabetes, and the dosage of contrast agents are critical risk factors for CI‐AKI.^15^ Therefore, for elderly patients, particularly those with chronic kidney disease or diabetes, a comprehensive assessment of the risk for CI‐AKI should be conducted prior to OCT‐guided PCI. It is essential to ensure that patients are adequately hydrated and to minimize the unnecessary use of contrast agents.

In conclusion, the OCTOBER study offers compelling evidence for the efficacy of OCT‐guided PCI in treating bifurcation lesions, while the ILUMIEN IV study underscores the capability of OCT‐guided PCI to achieve a larger MSA. However, the correlation between a larger MSA and improved clinical outcomes necessitates further investigation. Future research should focus on identifying patient populations that are more likely to benefit from this technique, particularly in relation to the characteristics of their coronary artery lesions. Given the tendency for coronary lesions in elderly patients to be more complex, the enhanced imaging resolution provided by OCT presents significant potential for optimizing PCI in this demographic. Furthermore, the use of OCT in bifurcation lesions involving the LMCA merits additional exploration, and the risk of CI‐AKI warrants increased attention.

## AUTHOR CONTRIBUTIONS

Yanwen Fang is responsible for writing the original manuscript, while Mengyue Yu oversees conceptualization, review, editing, and supervision.

## FUNDING INFORMATION

None.

## CONFLICT OF INTEREST STATEMENT

The authors disclosed no conflict of interest.

## References

[1] Ma L , Wang Z , Fan J , et al. Interpretation of report on cardiovascular health and diseases in China 2022. Chin Gen Pract. 2023;26(32):3975‐3994. doi:10.12114/j.issn.1007-9572.2023.0408

[2] Hong SJ , Kim BK , Shin DH , et al. Effect of intravascular ultrasound‐guided vs. angiography‐guided everolimus‐eluting stent implantation: the IVUS‐XPL randomized clinical trial. JAMA. 2015;314(20):2155‐2163. doi:10.1001/jama.2015.15454 26556051

[3] Zhang J , Gao X , Kan J , et al. Intravascular ultrasound versus angiography‐guided drug‐eluting stent implantation: the ULTIMATE trial. J Am Coll Cardiol. 2018;72(24):3126‐3137. doi:10.1016/j.jacc.2018.09.013 30261237

[4] Holm NR , Andreasen LN , Neghabat O , et al. OCT or angiography guidance for PCI in complex bifurcation lesions. N Engl J Med. 2023;389(16):1477‐1487. doi:10.1056/NEJMoa2307770 37634149

[5] Ali ZA , Landmesser U , Maehara A , et al. Optical coherence tomography‐guided versus angiography‐guided PCI. N Engl J Med. 2023;389(16):1466‐1476. doi:10.1056/NEJMoa2305861 37634188

[6] Ali ZA , Maehara A , Généreux P , et al. Optical coherence tomography compared with intravascular ultrasound and with angiography to guide coronary stent implantation (ILUMIEN III: OPTIMIZE PCI): a randomised controlled trial. Lancet. 2016;388(10060):2618‐2628. doi:10.1016/S0140-6736(16)31922-5 27806900

[7] Katagiri Y , De Maria GL , Kogame N , et al. Impact of post‐procedural minimal stent area on 2‐year clinical outcomes in the SYNTAX II trial. Catheter Cardiovasc Interv. 2019;93(4):E225‐E234. doi:10.1002/ccd.28105 30702187

[8] Madhavan MV , Gersh BJ , Alexander KP , Granger CB , Stone GW . Coronary artery disease in patients ≥80 years of age. J Am Coll Cardiol. 2018;71(18):2015‐2040. doi:10.1016/j.jacc.2017.12.068 29724356

[9] Tan Q , Wang Q , Liu D , Zhang S , Zhang Y , Li Y . Intravascular ultrasound‐guided unprotected left main coronary artery stenting in the elderly. Saudi Med J. 2015;36(5):549‐553. doi:10.15537/smj.2015.5.11251 25935174 PMC4436750

[10] de la Torre Hernandez JM , Baz Alonso JA , Gómez Hospital JA , et al. Clinical impact of intravascular ultrasound guidance in drug‐eluting stent implantation for unprotected left main coronary disease: pooled analysis at the patient‐level of 4 registries. JACC Cardiovasc Interv. 2014;7(3):244‐254. doi:10.1016/j.jcin.2013.09.014 24650399

[11] Ye Y , Yang M , Zhang S , Zeng Y . Percutaneous coronary intervention in left main coronary artery disease with or without intravascular ultrasound: a meta‐analysis. PLoS One. 2017;12(6):e0179756. doi:10.1371/journal.pone.0179756 28640875 PMC5481000

[12] Neumann FJ , Sousa‐Uva M , Ahlsson A , et al. 2018 ESC/EACTS guidelines on myocardial revascularization. EuroIntervention. 2019;14(14):1435‐1534. doi:10.4244/EIJY19M01_01 30667361

[13] Writing Committee Members , Lawton JS , Tamis‐Holland JE , et al. ACC/AHA/SCAI Guideline for Coronary Artery Revascularization: A Report of the American College of Cardiology/American Heart Association Joint Committee on Clinical Practice Guidelines. 2021 [published correction appears in J Am Coll Cardiol. 2022 Apr 19;79(15):1547. doi:10.1016/j.jacc.2022.03.330]. 34895950

[14] Amabile N , Rangé G , Souteyrand G , et al. Optical coherence tomography to guide percutaneous coronary intervention of the left main coronary artery: the LEMON study. EuroIntervention. 2021;17(2):e124‐e131. doi:10.4244/EIJ-D-20-01121 33226003 PMC9724912

[15] McCullough PA , Choi JP , Feghali GA , et al. Contrast‐induced acute kidney injury. J Am Coll Cardiol. 2016;68(13):1465‐1473. doi:10.1016/j.jacc.2016.05.099 27659469

